# Supplementary material for: Short-Stranded Zein Fibers for Muscle Tissue Engineering in Alginate-Based Composite Hydrogels
Source: Gels. 2023 Nov 17;9(11):914. doi: 10.3390/gels9110914 (PMC10671123; doi:10.3390/gels9110914)
Supplement: Supplementary file 1 [file gels-09-00914-s001.zip › gels-2689679-supplementary.pdf]

Supplementary Materials

Short-Stranded Zein Fibers for Muscle Tissue Engineering in Alginate-Based Composite Hydrogels

Lea Melzener [1,2], Sergio Spaans [2], Nicolas Hauck [2], André J. G. Pötgens [2], Joshua E. Flack [2], Mark J. Post [1,2] and Arın Doğan [2,*]

1 Department of Physiology, Maastricht University, Maastricht, The Netherlands
2 Mosa Meat B.V., Maastricht, The Netherlands
* Correspondence: arin@mosameat.com

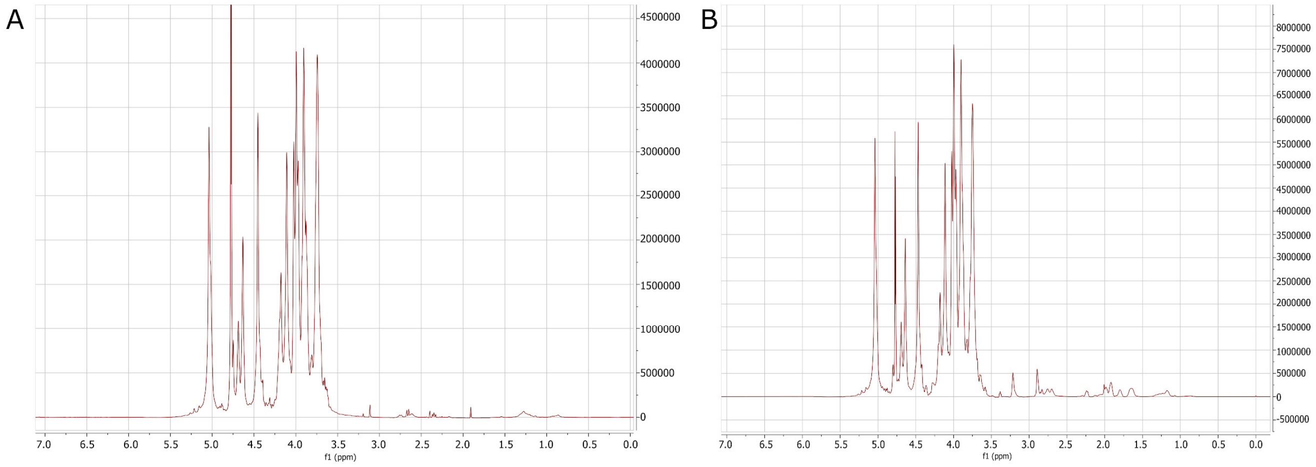

Figure S1. NMR spectra (**A**)Crude alginate before purification and functionalization. Some minor impurities were observed in the lower ppm range (1.0-3.5). (**B**) RGD-functionalized alginate. New peaks corresponding to the protons from the peptide side chains can be observed at the lower ppm range (1.0-3.5).

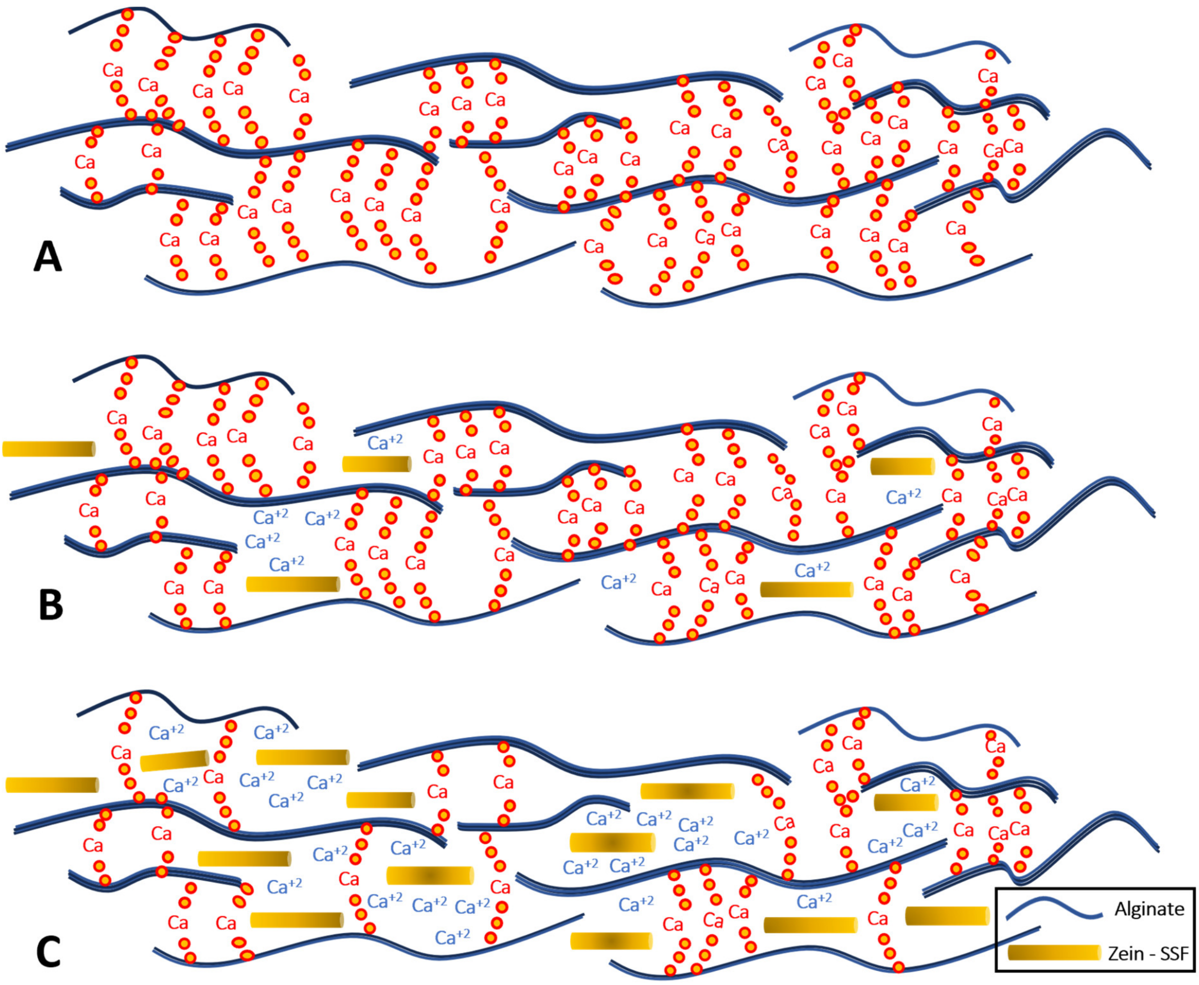

Figure S2. Zein interference of calcium crosslinking in alginate hydrogels**.** A: low concentration of zein-SSFs, with increased ionic crosslinking B: high concentration of zein-SSFs, with decreased ionic crosslinking.

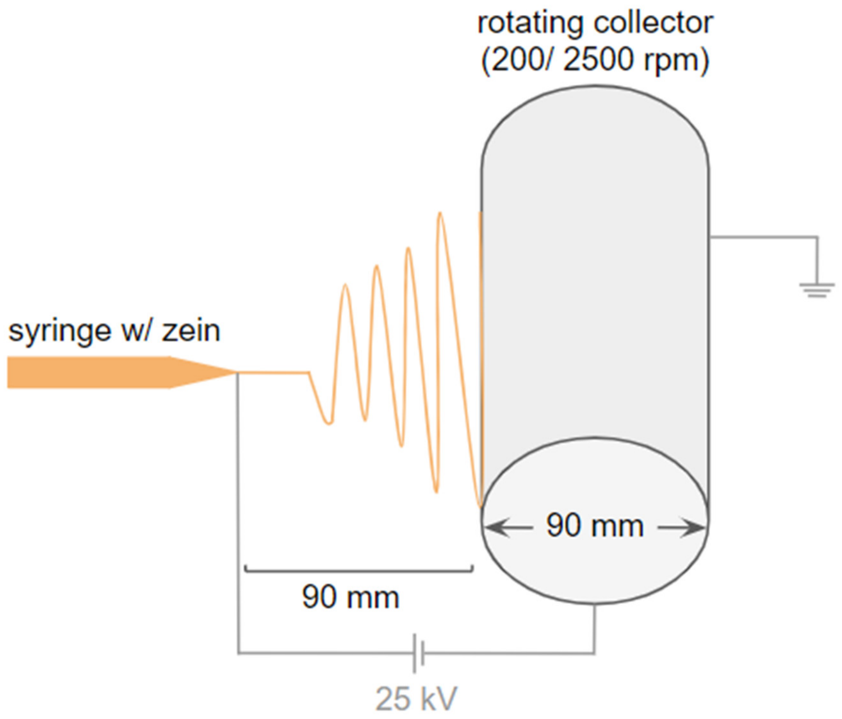

Figure S3. Electrospinning setup.

Table S1. Media formulations.

#	Component	Reference	Concentration
**Serum-free growth medium (SFGM)**			
1	DMEM/F-12	P04-041262B, PAN Biotech	
2	α-linolenic acid	L2376, Sigma Aldrich	1 µg ml^{-1}
3	bFGF-2	100-18B, Peprotech	10 ng ml^{-1}
4	bHGF	100-39H, Peprotech	50 ng ml^{-1}
5	Bovine Serum Albumin (BSA)	A9418, Sigma Aldrich	5 mg ml^{-1}
6	D-glucose	G7021, Sigma Aldrich	17.7 mM
7	Glutamax	35050061, ThermoFisher	2 mM
8	Hydrocortisone	H0888, Sigma Aldrich	36 ng ml^{-1}
9	IGF-1	100-11, Peprotech	100 ng ml^{-1}
10	ITSE	00-101, biogems	1%
11	L-ascorbic acid 2-phosphate (Vitamin C)	A8960, Sigma Aldrich	155 µM
12	LIF		5 ng ml^{-1}
13	PDGF-BB	100-14B, Peprotech	10 ng ml^{-1}
14	Penicillin/Streptomycin/Amphotericin (PSA)	17-745E, Lonza	1%
16	VEGF	100-20, Peprotech	10 ng ml^{-1}
**Serum-free myogenic differentiation medium (SFDM)**			
1	DMEM	A14430-01, Gibco	
2	EGF-1	AF-100-15, Peprotech	10 ng ml^{-1}
3	D-glucose	G7021, Sigma	5.5 mM
4	GlutaMax	35050061, ThermoFisher	2 mM
5	Human Serum Albumin	Rc HA NW20, Richcore Lifesciences	0.5 mg ml^{-1}
6	ITSE	00-101, biogems	2%
7	L-ascorbic acid 2-phosphate (Vitamin C)	A8960, Sigma Aldrich	40 µM
8	MEM Amino Acids Solution	11130-051, ThermoFisher	0.50%
9	$NaHCO_3$	P2256, Sigma Aldrich	6.5 mM
10	Penicillin/Streptomycin/Amphotericin (PSA)	17-745E, Lonza	1%
11	Soy hydrolysates	58903C, Merck	1%
12	Sodium l-lactate	71718, Sigma	10 mM
13	Sodium pyruvate	P2256, Sigma Aldrich	0.5 mM

Table S2. Antibodies used in this study.

Target	Colour	Source	Dilution	Reference	Application
**α**-alpha-actin-1	-	Abcam	1:5000	ab184705	Western Blot
**α**-actinin	-	Sigma-Aldrich	1:2500	A7811	Western Blot
desmin	-	Abcam	1:3000	ab227651	ELISA
f-actin	Atto550	Sigma-Aldrich	1:300	19083	IF
ITGA5	PE	Miltenyi Biotec	1:50	130-110-532	Flow
ITGA7	APC	Miltenyi Biotec	1:50	130-123-833	Flow
myoglobin	-	Abcam	1:2500	ab231725	Western Blot
myosin	-	Abcam	1.5 $\mu g\ ml^{-1}$	ab11083	ELISA
myosin	-	Abcam	0.8 $\mu g\ ml^{-1}$	ab197687	ELISA
streptavidin	HRP	Abcam	1:1000	ab7403	ELISA
anti-mouse	HRP	Dako	1:2000	P0447	Western Blot
anti-rabbit	HRP	Abcam	1:8000	ab6721	ELISA, Western Blot
